# Notes on the Characteristics of Aloes

**Published:** 1890-05

**Authors:** Walter M. James

**Affiliations:** Philadelphia


### NOTES ON THE CHARACTERISTICS OF ALOES.

Walter M. James, M. D., Philadelphia.

Head: Vertigo with constipation (Dunham). Vertigo with pain in right hypochondrium and at angle of escapula; icteric complexion and bilious urine. Dull, heavy headache with congestion of the liver; weight pressing down forehead to root of nose. A peculiar heavy, dull, pressing pain in the forehead, of no great severity, but which indisposes to, or even incapacitates for all exertion, particularly intellectual labor (P. P. Wells).

Dunham says that Aloes produces a singular combination of anxious restlessness, despondency, indisposition to mental or bodily exertion, and confusion of intelligence.

Aching above forehead with heaviness of eyes and nausea.

Falling out of the hair in adults.

Pressing headache; pressure extends to root of nose or to temples.

Throbbing headache.

Compelled to make eyes small during pain in forehead, to enable him to see. Dunham cured an obstinate headache by attention to this symptom.

Aggravations of head symptoms in early morning or in the evening or from sedentary habits.

Amelioration of head symptoms by cold water and the discharge of flatus.

Hypochondria: Pressure, tension, and discomfort in this region; a sensation of heat, pressure, and single severe stitches.

Abdomen distended with flatulence and there is fullness in the head and a sense of weight and dragging in the hypogastrium. Abdomen tender on pressure. Heaviness of abdomen extending to rectum and bladder.

Stool: Violent tenesmus, with stools of bloody water; great faintness during and after each stool. Stool at first very small, then more free, then follows a half-fluid, light yellow offensive stool and at last yellow, watery diarrhœa (Dunham).

The patient has frequent calls to stool which pass away in "gobs" either large or small, in consistency like jelly-fish, usually dark in color but sometimes colorless (G.). Sharp pains in the bowels with large quantities of flatus with the stool; when passing wind often has a stool. Stools in consistency like jelly-cakes; a quantity of clear, jelly-like substances, which may be green or white and adhere like congealed mucus (G.).

Sense of insecurity in the bowels, as if diarrhœa might occur at any time.

Stools lumpy and watery.

Diarrhœa, with want of confidence in the sphincter ani. The rectum seems full of fluid, which feels heavy as if it would fall out (M.).

Sense of weakness in rectum and anus, as if the latter would be suddenly relaxed in spite of the patient's will, and permit escaping fæces (Dunham).

Morning diarrhœa; colic, nausea, chilliness, sudden and irresistible desire for stool; can hardly get to the water-closet, before a dark, almost black, offensive, and liquid stool passes off. Fear lest stool should escape with flatus; feeling as if thin stool would escape when passing urine.

With the passage of stool feeling as if more were at hand. Urging to stool after eating; when rising after lying; from standing; when urinating.

Stool preceded and accompanied by much gurgling and rolling of flatus in the abdomen.

Diarrhœa brought on by eating, especially breakfast (Dunham).

Fear to pass flatus lest stool shall escape (Dunham).

Fear to urinate lest the slight bearing down involved will cause involuntary movement of the bowels (Dunham).

The same state of things occurs with the sphincter of the bladder as with the sphincter ani (Dunham).

On rising, he is obliged to run quickly to urinate; can hardly retain the urine.

Frequent desire to urinate.

Frequent desire to stool.

Dysentery, stools frequent and very painful with burning tenesmus at extremity of rectum.

Fistula in ano.

Diarrhœa ten A. M. and ten P. M., stool falling out almost unnoticed.

Diarrhœa continues only after ten A. M. (Dunham).

Diarrhœa with pain; soreness and burning in the rectum. Stools copious and watery with much flatus.

Great exhaustion and faintness after stools.

Driven out of bed at two or three o'clock in the morning for stool; can hardly rise quick enough.

Diarrhœa in early morning, five A. M. (Dunham).

Flatus burning and offensive.

Hemorrhoids with little fæces, bleeding often and profusely. They protrude like grapes; sore on wiping after stool.

PERINEUM: Sensation of a weight and feeling as if a plug were wedged in between the symphisis pubis and os coccygeus. Affection of prostate gland (Dunham). Sensation of a plug as above, with urging to stool.

PELVIS: Heat, weight, pressure, and dragging downward.

The symptoms which seem to me most characteristic are those of the head and abdomen, stool and urine. They are those on which my use of Aloes in practice has been based (Dunham).

GENITALS: Fullness and heaviness in the region of the uterus.

Pressing down in rectum during menses. Profuse menses.

Symptoms worse in hot, damp weather.

